# The relation between bulk (external) and internal measures of spinal stiffness

**DOI:** 10.1186/s12998-025-00615-x

**Published:** 2025-11-19

**Authors:** Casper Nim, Kenneth A. Weber II, Søren O’Neill, Rune Tendal Paulsen, Liam Culmsee-Holm, Evert Onno Wesselink, Yue-Li Sun, Peter Jun, Alexander Breen, Gregory N. Kawchuk

**Affiliations:** 1 Spine Centre of Southern Denmark, University Hospital of Southern Denmark, Sygehusvej 24, 6000 Kolding, Denmark; 2 Department of Regional Health Research, University of Southern Denmark, Campusvej 55, Odense, Denmark; 3 Division of Pain Medicine, Stanford University School of Medicine, Palo Alto, CA USA; 4 Longhua Hospital, Shanghai University of TCM, Shanghai, China; 5 Department of Physical Therapy, Faculty of Rehabilitation Medicine, University of Alberta, Edmonton, Canada; 6 Faculty of Science and Technology, Bournemouth University, Bournemouth, UK

**Keywords:** Bulk stiffness, Internal stiffness, Spinal stiffness, Measurements

## Abstract

**Background:**

While spinal stiffness is thought to be an important factor in the diagnosis and management of various spinal conditions, it is notoriously difficult to measure directly. As a result, clinicians often rely on posteroanterior palpation to estimate bulk stiffness as a proxy for the stiffness of internal spinal tissues. Unfortunately, the validity of this proxy remains uncertain. To investigate this, we posed two key research questions: (1) How do measurements of bulk stiffness correlate with direct measures of spinal stiffness? and (2) Can bulk stiffness measurements be normalized to more accurately reflect internal spinal stiffness?

**Methods:**

This cross-sectional measurement study investigated the relation between bulk and internal spinal stiffness in a young, asymptomatic cohort. Bulk stiffness defined as external resistance of the spine measured through mechanical indentation at the L3 vertebra, while internal spinal stiffness was assessed concurrently using fluoroscopic imaging. Linear regression was used to analyze the relation between bulk and internal spinal stiffness measures. Bulk measures were then normalized using physical measurements (e.g. height, weight) and tissue volume measures of the multifidi obtained by MRI (i.e. muscle volume) and reanalyzed.

**Results:**

Twenty-six persons (26) participated, with data from 7 of those being excluded due to fluoroscopic movement artifacts. Unnormalized bulk stiffness was found to correlate poorly with internal spinal stiffness (R^2^ = 0.1883). Normalization of bulk stiffness using factors such as body weight and multifidus muscle volume did not improve R^2^ values. Our results were further validated through post hoc analysis, suggesting *en bloc* movement of the lumbar spine.

**Conclusions:**

Raw bulk spinal stiffness values should not be used as a proxy for internal spinal stiffness as they measure unrelated constructs. Our results may help explain why bulk stiffness measures of the spine may not always align with clinical outcomes. Attempts to normalize bulk spinal stiffness to various human factors such as weight and paravertebral muscle volume did not improve the correlation between bulk spinal stiffness and internal spinal stiffness.

## Background

Tissue stiffness reflects the structural integrity of biological tissues and often changes due to pathology or injury [1]. For example, increased arterial wall stiffness is linked to cardiovascular disease [2], while reduced ligament stiffness may indicate injury or degeneration [3]. Similarly, spinal stiffness is a mechanical property that is a reflection of the structural connections between individual vertebrae. These connections are made primarily by ligaments (the intervertebral disc included), the facet joints, with additional contributions from other connecting tissues that include muscle in a non-contractile state [4]. Other inherent factors such as aging [5], pathology, or degenerative processes [6] may also contribute to spinal stiffness. Together, these factors create what can be conceptualized as the resting stiffness of the spine, the passive stiffness of the spine, or the internal stiffness of the spine. The internal stiffness of the spine, particularly in the posteroanterior translational direction, is thought to be clinically important as it is a reflection of the spine’s mechanical integrity [7].

In contrast, bulk stiffness of the spine can be found when a force is applied to the skin overlying the spine of a prone patient, and the resulting deformation appreciated [8]. This process can be performed manually (and subjectively) by a clinician or quantified objectively by an instrument. While this process captures the internal stiffness of the spine, it also captures additional elements, such as the stiffness of the skin, thoracolumbar fascia and spinal muscles. Evaluation of bulk spinal stiffness can also be susceptible to concurrent phenomena such as pain, respiration, paravertebral muscle contraction, changes in abdominal pressure, and the stiffness of the plinth [8–13].

How bulk spinal stiffness differs from internal spinal stiffness is of potential clinical importance as bulk spinal stiffness may vary independently of internal spinal stiffness. For example, changes in paravertebral volume will influence bulk spinal stiffness, but internal spinal stiffness may remain unchanged. Alternatively, changes in paravertebral composition through increased intramuscular fat (IMF) or dense and loose collagen (i.e. fibrosis) are speculated to contribute to densification, thickening, and stiffening of anatomical structures affecting both bulk and internal spinal stiffness. Although these two conceptualizations of stiffness may each have clinical value, bulk spinal stiffness may not serve as a reliable proxy for internal spinal stiffness or for guiding clinical decisions based on it. Because internal stiffness remains difficult to assess directly in clinical settings, bulk stiffness is often used instead as a surrogate for spinal mechanical behavior. However, this substitution may help explain why bulk stiffness measures do not consistently align with clinical outcomes [14, 15].

Given the above, we devised a study in which bulk spinal stiffness and internal spinal stiffness are collected concurrently using instrumentation (mechanical indentation and fluoroscopy, respectively) to allow for direct comparison. This design allowed us to pose several novel research questions. First, how do measurements of bulk stiffness correlate with direct measures of spinal stiffness and second, can bulk stiffness measurements be refined to more accurately reflect internal spinal stiffness?

## Methods

### Design

This was a cross-sectional measurement study that included participants without back pain, in which spinal stiffness, conceptualized as both bulk and internal stiffness, was assessed using two complementary measurement approaches. The study was approved by the Health Science Ethics Committee of the Region of Southern Denmark (ID S-20190094).

### Participants

Participants without back pain were recruited from the Masters Program of Clinical Biomechanics, University of Southern Denmark. Inclusion criteria consisted of no previous back surgery, ability to understand verbal and written Danish, no pregnancy or suspicion of pregnancy, no psychological disorders, body mass index (BMI) < 30, and no known rheumatological diseases (e.g. connective tissue disorders). Exclusion criteria included intolerance of test procedures (e.g., procedure-related pain or inability to hold breath during testing), or data collection difficulties that could influence data quality/integrity.

Potential participants were recruited through the University of Southern Denmark's internal digital communication platform (*e-learn*) and verbal advertising of the project in the classroom. Written project information was provided for those who expressed an interest in participation, and in accordance with the ethical requirements, several days were allowed for recruits to consider their participation. Informed, written consent was obtained from all participants. Participants had the option to withdraw their consent to take part in the study at any time.

### Protocol overview

**Fig. 1:**
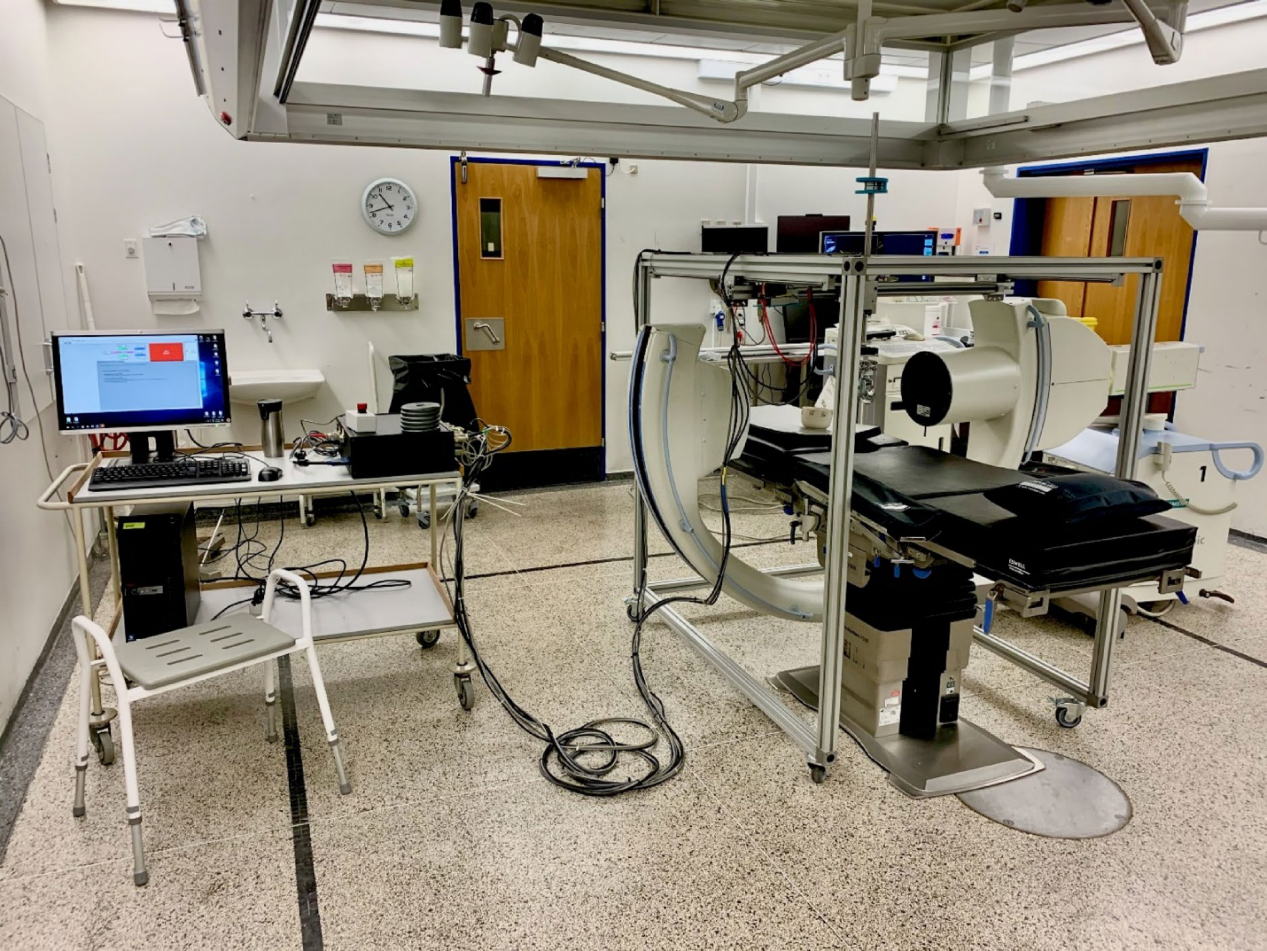
Experimental setup. The mechanical indentation device pictured inside of the fluoroscopic unit

Participants were asked to lie prone on a plinth positioned inside a fluoroscopic unit. A mechanical indentation device, suspended by an aluminum gantry, was positioned over the third lumbar vertebra (L3) of the prone participant (Fig. 1). Prior to loading, the indenter head was positioned so that it lightly touched the skin, establishing the zero-displacement reference point. The indentation device applied discrete posteroanterior loads via the addition of weights with a nominal mass of 1 kg each, up to a maximum load of 60 N [16]. During this loading, continuous fluoroscopic imaging was used to record the displacement of the lumbar vertebrae.

Bulk stiffness was defined as the maximal applied load divided by the associated displacement measured by the indentation device. Internal spinal stiffness was calculated as the same applied load divided by the displacement of the L3 vertebra as visualized by fluoroscopy.

Prior to testing, participants also underwent magnetic resonance imaging (MRI) to assess lumbar paravertebral muscle volume and intervertebral disc degeneration.

### Bulk stiffness and indentation

Bulk stiffness was determined using, a mechanical indentation device mounted on an aluminum gantry (Width: 1080 mm; Height: 1090 mm; Length: 1510 mm) that applies posteroanterior loads through a rolling indenter head positioned above the L3 spinous process. The indenter head is vertically aligned to the target using an integrated laser (GLX Laser Site, Barska, USA), and loads are applied incrementally using 1 kg weight plates (~ 10N), up to a maximum of ~ 60 N. Indentation was performed in static mode, meaning the indenter head was manually positioned over L3 with no movement in the horizontal plane.

The applied load is transferred along the Z-axis (vertical/posteroanterior) and the resulting tissue deformation is measured using a string potentiometer (TE Connectivity, USA; resolution = 0.020 mm). Stepper motors control movement in the X, Y, and Z directions (www.stepperonline.com, China; resolution = 0.007 mm), though only the Z-axis was used in this study. All displacement and load data were acquired through custom LabVIEW software (National Instruments, USA), which also displayed real-time force–displacement curves and allowed for data export. The indentation device has demonstrated high precision at all loads and displacements with minimal systematic measurement bias [17].

### Internal stiffness and fluoroscopy

In this study, internal spinal stiffness was calculated from the applied load at L3 versus the visualized displacement of the L3 vertebra as determined by concurrent fluoroscopy (Ziehm Vision FD C-arm system, Ziehm Imaging, Nuremberg, Germany). Although indentation was performed only at L3, the fluoroscopic imaging captured displacements across L2-L4, allowing for an assessment of how motion was distributed throughout the lumbar spine. The resulting images were then input into custom image analysis software written in MATLAB (R2022b,—The Mathworks Inc., Natick, MA, USA) to quantify any change in position within the sagittal imaging plane. This system [18] uses a cross-correlation method to track the vertebra between image frames and has been demonstrated to be both accurate and repeatable in measuring the translation of vertebrae [19]. Following tracking the vertebral bodies, the specific displacement values used for internal stiffness calculations were derived by quantifying the movement of reference points on the posterior border of the vertebral body, namely its midpoint. To convert vertebral displacement from pixels (image resolution: 512 × 512, Unit8) to millimeters, radiopaque beads (3.18 mm diameter) embedded in the indentation device were utilized. The area of each visible bead in the fluoroscopic field was measured using ImageJ software (National Institutes of Health, USA) and mathematically converted to a diameter in pixels. This pixel diameter, along with the known physical diameter of the bead, was used to establish a pixels-to-millimeters conversion factor for each participant. Given that the beads were positioned over the spinous process, magnification distortion between the beads and the vertebral bodies was assumed to be negligible. In addition to continuous fluoroscopy during indentation, static fluoroscopic images were taken at various load increments, including specifically at the minimal (0N) and the maximum (60N) indentation loads used for stiffness calculation. The displacement of L3 between these minimal and 60N maximal load images was used for determining internal spinal stiffness (Table 1).

**Table 1:** Characteristics of participants

Sex	Age (yr)	Weight (kg)	Height (m)	Abdominal circumference (m)	BMI (kg/m^2^)	Mean muscle volume (ml)
Male	25	85.50	1.91	0.87	23.44	150.10
Male	24	76.70	1.90	0.79	21.25	143.70
Female	25	80.00	1.81	0.84	24.42	106.70
Female	25	78.80	1.72	0.92	26.64	83.45
Male	25	79.50	1.79	0.89	24.81	103.85
Female	25	62.50	1.70	0.79	21.63	94.70
Female	26	56.00	1.62	0.73	21.34	91.00
Male	26	96.80	1.86	0.94	27.98	149.10
Male	26	95.40	1.90	0.93	26.43	124.85
Male	26	66.40	1.70	0.77	22.98	112.65
Female	23	69.00	1.71	0.73	23.60	107.90
Female	26	68.60	1.74	0.84	22.66	103.75
Male	26	86.20	1.93	0.88	23.14	140.05
Male	26	85.20	1.91	0.90	23.35	142.90
Female	25	68.00	1.70	0.77	23.53	105.95
Male	26	73.20	1.82	0.85	22.10	123.20
Male	28	60.70	1.74	0.78	20.05	92.95
Male	26	96.70	1.91	0.85	26.51	159.40
Female	26	67.80	1.81	0.87	20.70	108.00

### MRI

**Fig. 2:**
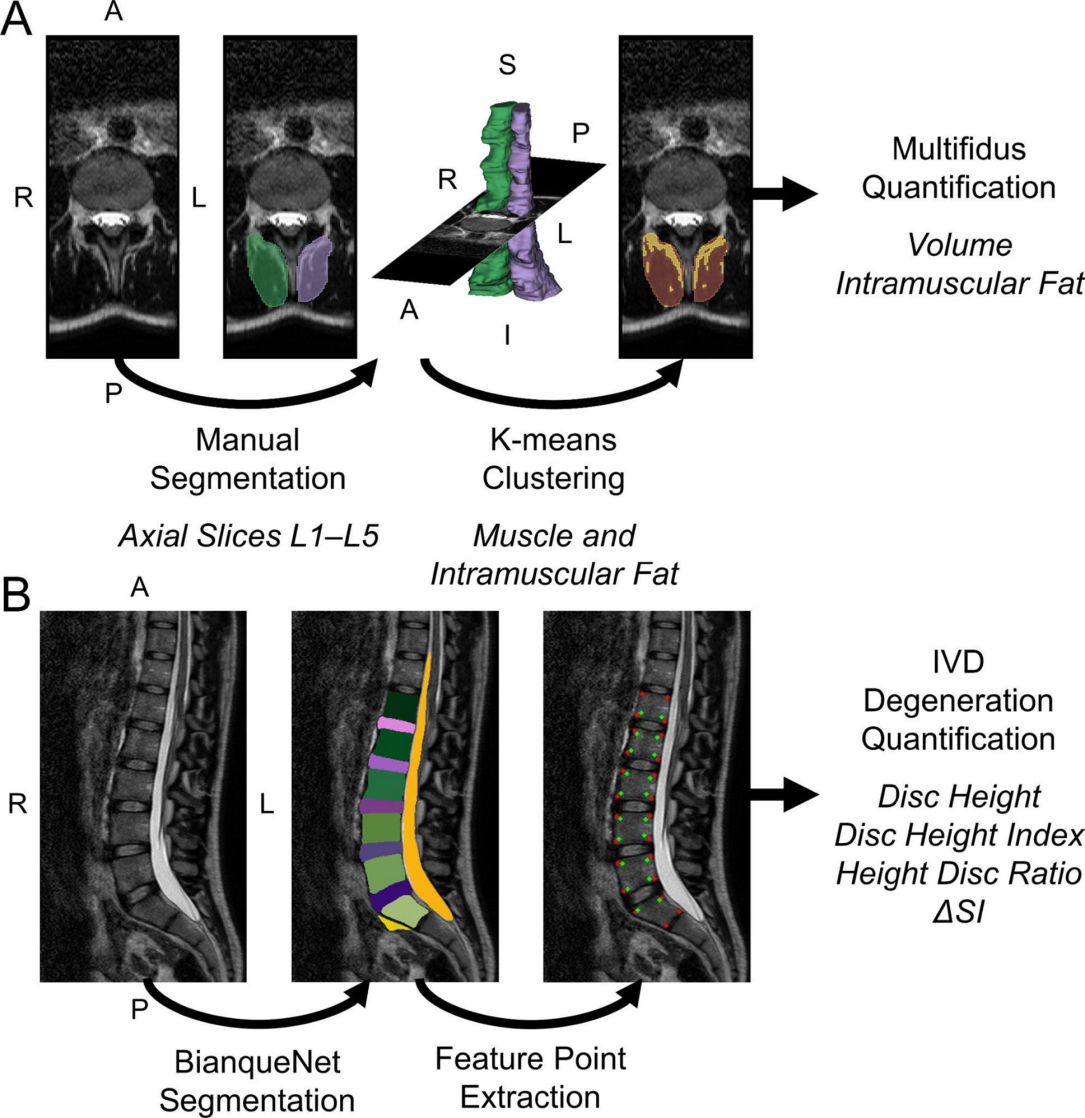
Assessment of muscle and intervertebral discs. Using T_2_-weighted images of lumbar spine, the lumbar multifidus muscles and intervertebral discs were quantitatively assessed. **A** The left and right lumbar multifidi were manually segmented on each axial slice between the superior endplate of the L1 vertebra and the inferior endplate of the L5 vertebra. K-means clustering was applied to separate muscle from fat and measures of multifidus muscle volume and intramuscular fat were extracted. **B** The midsagittal image was input into an automated open-source deep-learning model, BianqueNet, to assess intervertebral disc degeneration. Disc height, disc height index, disc height-to-diameter ratio, and a measure of water loss, the signal-intensity peak-deviation degree from the center (ΔSI), were extracted

To assess whether paravertebral muscle morphometry is associated with internal/external stiffness, we utilized MRI to quantify paravertebral muscle volume and IMF. Lumbar spine MRI was performed supine using a 1.5 T Philips Achieva scanner equipped with a 15-channel SENSE spine coil and a T_2_-weighted VISTA spin-echo sequence (repetition time = 2000 ms, echo time = 120 ms, echo train length = 97, bandwidth = 617 Hz, field of view = 73 × 560 × 560, resolution = 1.00 mm × 0.58 mm × 0.58 mm) to extract values on lumbar multifidus volume and IMF as well as lumbar intervertebral disc degeneration [20]. To assess the multifidus, an experienced, blinded rater (EOW) first manually segmented the left and right lumbar multifidus on each axial slice between the superior endplate of the L1 vertebra and inferior endplate of the L5 vertebra using 3D Slicer following anatomical cross-references as previously defined [21, 22]. K-means clustering (number of clusters = 2, initialization method = k-means++, number of initializations = 50) was applied to separate muscle from fat across the left and right multifidus. Multifidus muscle volume (muscle volume + IMF volume) and percent IMF (IMF volume/(IMF volume + muscle volume) × 100) was extracted for the left and right muscles and then averaged (Fig. 2A) [23]. To assess intervertebral disc degeneration, the midsagittal image was input into an open-source deep-learning model, BianqueNet, to automatically identify the lumbar vertebrae and intervertebral discs, sacrum, presacral fat area, cerebrospinal fluid followed by feature point extraction. Intervertebral disc degeneration at the L1-L2, L2-L3, L3-L4, L4-L5, and L5-S1 disc levels was quantified using the disc height (DH), disc height index (DHI), disc height-to-diameter ratio (HDR), and a measure of water loss, the signal-intensity peak-deviation degree from the center (ΔSI) (Fig. 2B) [24]. MRI analyses were performed in a Python environment using Pytorch, SciPy, and scikit-learn [25–27].

### Statistical analysis

Individual participant characteristics were presented for each variable, and descriptive statistics for bulk spinal stiffness and internal spinal stiffness were calculated using median and interquartile range (IQR).

A simple linear regression was performed to determine the relation between two variables (e.g. bulk spinal stiffness vs. internal spinal stiffness). The regression was performed by fitting a linear equation to the observed data. The resulting Coefficient of Determination (R^2^) indicated the proportion of the variance in the dependent variable that is predictable from the independent variable. All analyses were completed in Microsoft Excel for Windows 11.Normalization is a process used to place one measure in relation to another and is the same approach used to portray a person’s weight as a function of their height (i.e. BMI). Similarly, we nominated several anthropometric normalizing factors that were relatively easy to obtain including weight, height, BMI, and abdominal circumference (ABC). In addition, we also included multifidus volume measures obtained by MRI as the gold standard factor for normalization. To explore whether any normalization factor improved the association between bulk and internal stiffness, bulk stiffness was divided by each factor and the resulting values were visually and statistically compared using R^2^ as an indicator of explanatory strength. The resulting normalized values of bulk spinal stiffness were then compared again to the internal spinal stiffness through simple linear regression. We additionally utilized MRI to investigate if the degenerative status of the lumbar spine was a factor in our analysis.

## Results

### Participants

We tested consenting participants from October 2019 to February 2020 who were between 18–35 years of age without current back pain and no history of back pain in the preceding 3 months. Twenty-six [26] participants took part in the study. Of these, data was excluded from 7 participants due to anomalies in the fluoroscopic imaging arising from breathing and/or movement. These artifacts compromised the ability to reliably quantify vertebral displacement, and exclusion was necessary to ensure the validity of the motion tracking and stiffness estimates. The 19 remaining participants ranged in age from 23 to 28 years (mean 25.2), with 7 self-identifying as women and 12 as men.

### Spinal stiffness

The median bulk spinal stiffness was 4.40 N/mm (IQR = 0.97), while the median internal spinal stiffness was 9.27 N/mm (IQR = 2.91).

### Disc degeneration

MRI-based assessment of disc degeneration was reviewed within the research team, including clinician researchers experienced in spinal imaging and degenerative grading. Most participants had no or minimal degeneration, limiting its relevance for further analysis.

### Comparison of bulk spinal stiffness and internal spinal stiffness values

**Fig. 3:**
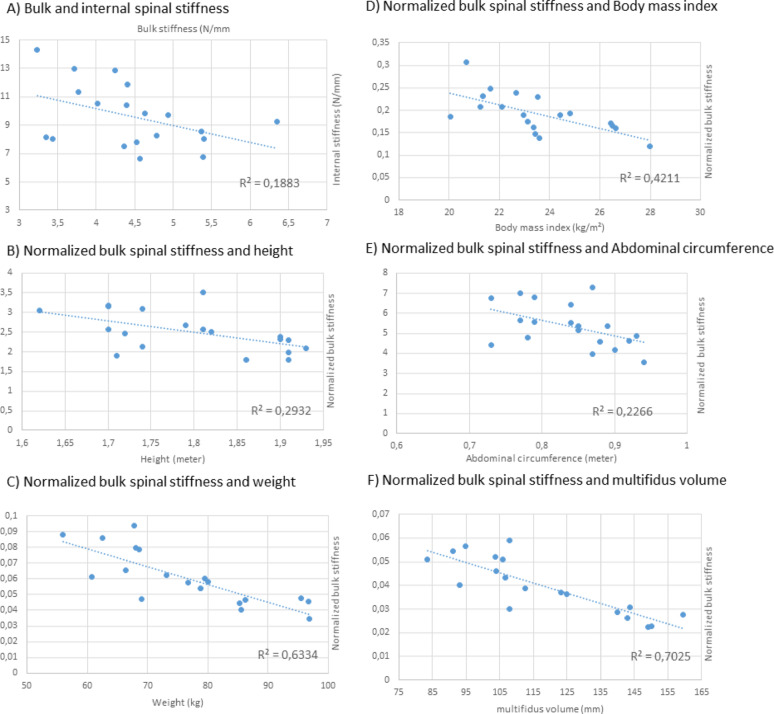
Associations between bulk spinal stiffness and internal spinal stiffness, and between normalized bulk stiffness and anthropometric or anatomical variables

Within-participant comparison of unnormalized bulk spinal stiffness to internal spinal stiffness values for the 19 full-data participants resulted in a coefficient of determination (R^2^) of 0.1883 (Fig. 3A).

### Normalization factors

The R^2^ values of the normalized bulk spinal stiffness values in relation to the normalizing value were as follows: height (0.2932), weight (0.6334), BMI (0.4211), ABC (0.2266), average multifidus volume (0.7025) (Fig. 3B–F). As bulk spinal stiffness normalized to weight performed almost as well as our gold standard (multifidis volume), and is much easier and cheaper to acquire, we used both these normalization factors to re-evaluate the relation between bulk stiffness and internal spinal stiffness.

### Comparison of normalized bulk spinal stiffness and internal spinal stiffness:

When bulk spinal stiffness values normalized by weight were compared against internal spinal stiffness, the resulting R^2^ value (0.0712) was lower than the R^2^ value obtained when comparing unnormalized bulk spinal stiffness values to internal spinal stiffness values (0.1883). Similarly, bulk stiffness values normalized by mean muscle volume (our gold standard) were compared to internal spinal stiffness values, the R^2^ value was poor (0.0355).

### Post HOC analysis

**Fig. 4:**
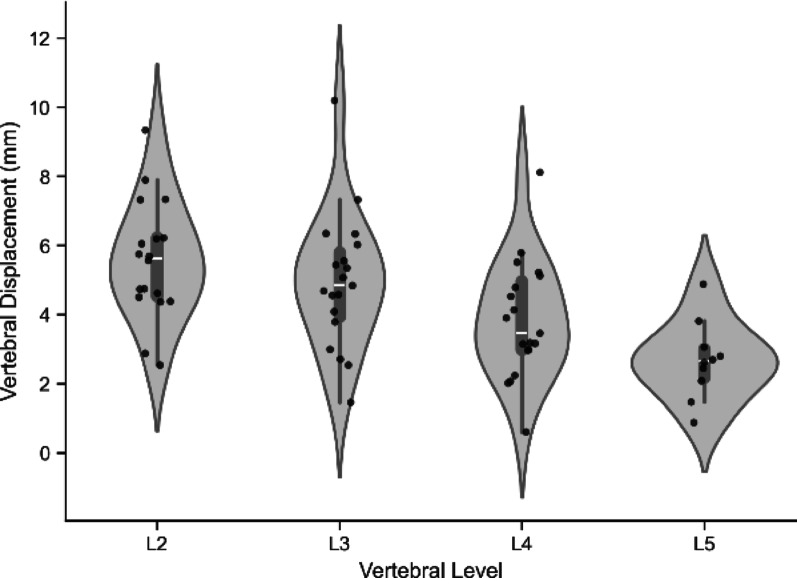
Displacement across all visible vertebrae

To further explore the disconnect between bulk and internal spinal stiffness, we conducted an additional post hoc analysis focused on vertebral motion. Although loading was applied only at the L3 vertebra, fluoroscopic imaging captured motion across L2-L5. O*f the 19 participants L2 was trackable 18 times, L3 and L4 19 times, and L5 10 times*. An analysis of all visible vertebrae confirmed a cranial-to-caudal gradient in displacement. The median (IQR) displacement was greatest at the most cranial levels (L2: 5.6 (1.7) mm) and progressively decreased towards the pelvis (L3: 4.8 (1.8) mm; L4: 3.5 (2.0) mm; L5: 2.7 (0.8) mm). This pattern further supports the *en bloc* movement of the lumbar spine (Fig. 4).

**Fig. 5:**
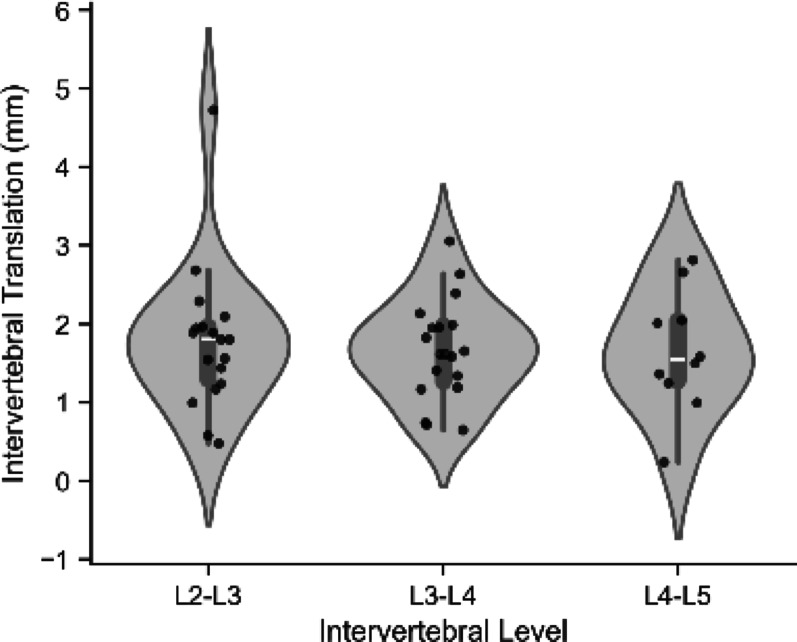
Translation across all visible vertebral segments

We then quantified the relative slip between adjacent vertebrae (intervertebral translation of vertebral segments) to determine if the spine was deforming internally. The median (IQR) intervertebral range of translation over the indentation was for L2-L3: 1.8 (0.7) mm, L3-L4: 1.6 (0.7) mm and L4-L5 1.5 (0.8) mm. Critically, while small these translations are greater than the threshold of measurement error 1.1 mm standard error of measurement previously established for this quantitative fluoroscopy method [19] (Fig. 5).

Taken together, these findings suggest that the lumbar vertebrae moved largely together (en bloc). The displacement pattern, with cranial levels moving the most, is consistent with the behavior of a rigid segment being displaced between the pelvis and the semi-compressible rib cage. This indicates that the measured "internal" displacement may reflect larger-scale system mechanics rather than true, localized intervertebral joint motion.

Finally, we evaluated whether vertebral displacement response to load was associated with common anthropometric variables. Linear regression showed poor associations with height (R^2^ = 0.0299), weight (R^2^ = 0.0462), BMI (R^2^ = 0.0337), and mean multifidus volume (R^2^ = 0.0399), reinforcing the idea that internal spinal mechanics are not well captured by external characteristics.

## Discussion

In this study, we investigated the relation between external measures of spinal stiffness (bulk spinal stiffness) and the actual, internal stiffness of the spine itself (internal spinal stiffness). While internal spinal stiffness is a constituent part of a bulk spinal stiffness measurement, additional factors that make up bulk spinal stiffness (e.g., skin stiffness, paravertebral compressibility) result in a low correlation between these two measures. Attempts to normalize bulk spinal stiffness to various human factors such as weight and paravertebral muscle volume did not improve the correlation between bulk spinal stiffness and internal spinal stiffness. Post-hoc analysis of our data provided additional evidence in support of the primary data.

Our results indicates that bulk spinal stiffness should not be considered as a proxy for the internal, resting, or actual stiffness of the spine in the postero-anterior direction, especially when making clinical decisions about the status of internal spinal stiffness or treatment directed at altering internal spinal stiffness [28]. One way to conceptualize this disconnect between bulk spinal stiffness and internal spinal stiffness is an analogy of measuring general function using self-reported measures (e.g. Oswestry Disability Index) and objective measures (e.g. Sit-to-Stand test) [29]. Both provide important information, but neither should be used as a surrogate for the other.

Our fluoroscopic results provide new insight into how the lumbar spine responds to posteroanterior loading. Consistent with previous reports using dynamic MRI [30], we observed that loading applied at a single vertebra produced motion across multiple levels, indicating that the lumbar spine behaves as an integrated unit rather than as independent segments. In our sample, displacement decreased progressively from cranial to caudal levels, suggesting that the semi-rigid rib cage acts as a constraint while the pelvis provides a fixed base, leading to greater motion in the more cranial vertebrae. This has also been shown in more clinical studies using the VerteTrack [31].

Intervertebral translations were present but small, only slightly above the threshold of measurement error, reinforcing the impression of predominantly *en bloc* motion with limited relative deformation between vertebrae. Taken together, these findings suggest that the majority of the displacement captured by fluoroscopy reflects large-scale system mechanics (ribcage-to-pelvis bending), consistent with an *en bloc* response to posteroanterior loading. Limited intervertebral translations were induced during the static press, ranging from just 1.5 to 1.8 mm. Although small, these values were reliably detected, being greater than the error threshold for quantitative fluoroscopy, and are comparable to normative ranges reported in healthy populations during flexion tasks [19, 32, 33]. Importantly, these small translations did not appear to concentrate at the site of indentation, further supporting the view that the response is dominated by system-level rather than localized mechanics. Thus, the “internal” displacement we measured is better understood as an expression of regional lumbar motion, with only a minor component of true segmental translation. Recognising this distinction helps reconcile the difference between bulk (external) and internal measures of spinal stiffness. This finding is perhaps critical in attempting to understand why validation studies that asses stiffness using manual palpation fail [34–36] and why treatments aimed at specific spinal structures have little impact on patient-reported outcomes [37–40].

Importantly, our post hoc analysis showed that there are measures that vary with bulk spinal stiffness but are unrelated to internal spinal stiffness and they can fluctuate independently of internal spinal stiffness; a finding that supports our original premise that bulk spinal stiffness contains elements independent of internal spinal stiffness that make it an inappropriate proxy for internal spinal stiffness.

While our results do not suggest normalized bulk stiffness values can act as a proxy for measures of internal spinal stiffness, normalized bulk stiffness measures may have importance elsewhere. Specifically, when comparing stiffness between patients, one can imagine that the spinal tissues of a child will move proportionally more than when the same force is applied to the spine of an adult. To make these between-patient comparisons more meaningful, normalization could mitigate such an effect.

### Limitations

Several limitations should be acknowledged when interpreting this study's findings. First, our participant cohort was relatively young and homogeneous, consisting of university students without back pain and degenerative changes, which may limit the generalizability of the results to older populations or those with existing low back conditions. Importantly though, this homogeneity of disc morphology may have been helpful in our analysis as degeneration status was not a potential confounder. Second, while our concurrent use of mechanical indentation and fluoroscopy aimed to reduce measurement variation, both methods have inherent sampling limitations. Furthermore, seven participants were excluded from the study due to movement artifacts in fluoroscopic images. These technical limitations, particularly in relation to participant movement, highlight the need for more robust image acquisition techniques in future studies. Third, we assessed intramuscular fat using T_2_-weighted images, which only demonstrates moderate reliability with the current reference standard of Dixon fat–water MRI [23]. Additionally, we limited imaging of muscle to the lumbar multifidus due to the restricted field-of-view and did not include other muscles that can contribute to spinal stiffness. Also, the assessment of disc degeneration was two-dimensional and limited to the midsagittal image. A three-dimensional assessment of disc degeneration may have provided more sensitive measures of disc degeneration [24]. Therefore, the assessment of the lumbar paraspinal muscles and intervertebral discs with MRI could be improved. The normalization process used in this study, while necessary for comparison, may introduce additional variability. Although we selected participant characteristics like BMI and muscle volume to normalize stiffness measures, it is possible that these factors do not fully account for inter-individual variability in spinal stiffness, particularly in more heterogeneous populations. Future studies should explore this further. Finally, fluoroscopy is not a feasible option in routine clinical practice, future research should explore non-invasive methods that can approximate internal spinal stiffness more directly.

## Conclusions

Raw bulk spinal stiffness values should not be used as a proxy for internal spinal stiffness, as they represent different conceptualizations of the spinal stiffness construct. Our findings may help explain why bulk stiffness measures do not consistently align with clinical outcomes. Attempts to normalize bulk stiffness to anthropometric factors such as body weight or paravertebral muscle volume did not improve its association with internal spinal stiffness. There remains an unmet need to provide clinicians with direct information about the resting, passive internal stiffness of the spine to support clinical decision-making.

## Data Availability

Data, including anonymized MRIs, are available upon reasonable request, please contact Casper Nim (casper.nim@rsyd.dk).

## Abbreviations

ABC: Abdominal circumference
BMI: Body mass index
IMF: Intramuscular fat
MRI: Magnetic resonance imaging

## Declarations

### Generative AI and AI-assisted technologies in the writing process

During the preparation of this work, the authors used ChatGPT 4o and GrammarlyGO to assess paraphrasing, optimize grammar, and proofread. After using this tool, the authors reviewed and edited the content as needed and take full responsibility for the content of the publication.

## References

[1] Wells RG. Tissue mechanics and fibrosis. Biochim Biophys Acta. 2013;1832(7):884–90. 23434892 10.1016/j.bbadis.2013.02.007 PMC3641165

[2] Mitchell GF, Hwang SJ, Vasan RS, Larson MG, Pencina MJ, Hamburg NM, m.fl. Arterial stiffness and cardiovascular events. Circulation. 2010;121(4):505–11. 10.1161/CIRCULATIONAHA.109.886655 PMC2836717 20083680

[3] Woo SLY, Debski RE, Zeminski J, Abramowitch SD, Serena S. Chan Saw MS, Fenwick JA. Injury and repair of ligaments and tendons. Ann Rev Biomed Eng. 2000;2:83–118. 10.1146/annurev.bioeng.2.1.83 11701508

[4] Han KS, Rohlmann A, Kim K, Cho K, Kim YH. Effect of ligament stiffness on spinal loads and muscle forces in flexed positions. Int J Precis Eng Manuf. 2012;13:2233–8.

[5] Iida T, Abumi K, Kotani Y, Kaneda K. Effects of aging and spinal degeneration on mechanical properties of lumbar supraspinous and interspinous ligaments. Spine J. 2002;2(2):95–100. 14588267 10.1016/s1529-9430(02)00142-0

[6] Ellingson A, Shaw M, Giambini H, An K. Comparative role of disc degeneration and ligament failure on functional mechanics of the lumbar spine. Comput Methods Biomech Biomed Eng. 2016;19:1009–18. 10.1080/10255842.2015.1088524 PMC4808500 26404463

[7] Wong AYL, Kawchuk GN. The clinical value of assessing lumbar posteroanterior segmental stiffness: a narrative review of manual and instrumented methods. PM&R. 2017;9(8):816–30. 27993736 10.1016/j.pmrj.2016.12.001

[8] Kawchuk GN, Fauvel OR. Sources of variation in spinal indentation testing: indentation site relocation, intraabdominal pressure, subject movement, muscular response, and stiffness estimation. J Manipulative Physiol Ther februar. 2001;24(2):84–91. 10.1067/mmt.2001.112566 11208220

[9] Hodges PW, Eriksson AEM, Shirley D, Gandevia SC. Intra-abdominal pressure increases stiffness of the lumbar spine. J Biomech. 2005;38(9):1873–80. 16023475 10.1016/j.jbiomech.2004.08.016

[10] Stanton T, Kawchuk G. The effect of abdominal stabilization contractions on posteroanterior spinal stiffness. Spine (Phila Pa 1976). 2008;33(6):694–701. 10.1097/BRS.0b013e318166e034 18344865

[11] Edgecombe TL, Kawchuk GN, Long CR, Pickar JG. The effect of application site of spinal manipulative therapy (SMT) on spinal stiffness. Spine J. 2015;15(6):1332–8. 24139864 10.1016/j.spinee.2013.07.480 PMC3989461

[12] Stanton TR, Moseley GL, Wong AYL, Kawchuk GN. Feeling stiffness in the back: a protective perceptual inference in chronic back pain. Sci Rep 29. 2017 https://www.ncbi.nlm.nih.gov/pmc/articles/PMC5575135/10.1038/s41598-017-09429-1 PMC5575135 28851924

[13] Wong AYL, Parent EC, Prasad N, Huang C, Chan KM, Kawchuk GN. Does experimental low back pain change posteroanterior lumbar spinal stiffness and trunk muscle activity? A randomized crossover study. Clin Biomech (Bristol, Avon). 2016;34:45–52. 10.1016/j.clinbiomech.2016.03.006 27064671

[14] Nim CG, Kawchuk GN, Schiøttz-Christensen B, O’Neill S. Changes in pain sensitivity and spinal stiffness in relation to responder status following spinal manipulative therapy in chronic low Back pain: a secondary explorative analysis of a randomized trial. BMC Musculoskel Disord. 2021;22(1):23. 10.1186/s12891-020-03873-3 PMC7786943 33407345

[15] Wong AYL, Parent EC, Dhillon SS, Prasad N, Kawchuk GN. Do participants with low back pain who respond to spinal manipulative therapy differ biomechanically from nonresponders, untreated controls or asymptomatic controls? Spine. 2015;40(17):1329–37. 26020851 10.1097/BRS.0000000000000981

[16] Hadizadeh M, Kawchuk G, French S. A consensus approach toward the standardization of spinal stiffness measurement using a loaded rolling wheel device: results of a Delphi study. BMC Musculoskel Disord. 2021;22:436. 10.1186/s12891-021-04313-6 PMC8120899 33985464

[17] Brown BT, Blacke A, Carroll V, Graham PL, Kawchuk G, Downie A, m.fl. The comfort and safety of a novel rolling mechanical indentation device for the measurement of lumbar trunk stiffness in young adults. Chiropractic & Manual Therapies. 2017;25(1). 10.1186/s12998-017-0153-z 10.1186/s12998-017-0153-z PMC5541409 28785399

[18] Muggleton JM, Allen R. Automatic location of vertebrae in digitized videofluoroscopic images of the lumbar spine. Med Eng Phys. 1997;19(1):77–89. 9140876 10.1016/s1350-4533(96)00050-1

[19] Breen A, Breen A. Accuracy and repeatability of quantitative fluoroscopy for the measurement of sagittal plane translation and finite centre of rotation in the lumbar spine. Med Eng Phys. 2016;38(7):607–14. 27129784 10.1016/j.medengphy.2016.03.009

[20] Mugler JP. Optimized three-dimensional fast-spin-echo MRI. J Magn Reson Imag. 2014;39(4):745–67. 10.1002/jmri.24542 24399498

[21] Fedorov A, Beichel R, Kalpathy-Cramer J, Finet J, Fillion-Robin JC, Pujol S, m.fl. 3D Slicer as an image computing platform for the quantitative imaging network. Magn Reson Imag. 2012;30(9):1323–41. 10.1016/j.mri.2012.05.001 PMC3466397 22770690

[22] Crawford RJ, Cornwall J, Abbott R, Elliott JM. Manually defining regions of interest when quantifying paravertebral muscles fatty infiltration from axial magnetic resonance imaging: a proposed method for the lumbar spine with anatomical cross-reference. BMC Musculoskelet Disord. 19. 2017;18(1):25. 10.1186/s12891-016-1378-z PMC5247810 28103921

[23] Wesselink EO, Elliott JM, Pool-Goudzwaard A, Coppieters MW, Pevenage PP, Di Ieva A, m.fl. Quantifying lumbar paraspinal intramuscular fat: Accuracy and reliability of automated thresholding models. N Am Spine Soc J. 2024;17:100313. 10.1016/j.xnsj.2024.100313 PMC10869289 38370337

[24] Zheng HD, Sun YL, Kong DW, Yin MC, Chen J, Lin YP, m.fl. Deep learning-based high-accuracy quantitation for lumbar intervertebral disc degeneration from MRI. Nat Commun. 2022;13(1):841. 10.1038/s41467-022-28387-5 PMC8837609 35149684

[25] Ansel J, Yang E, He H, Gimelshein N, Jain A, Voznesensky M, m.fl. PyTorch 2: Faster machine learning through dynamic python bytecode transformation and graph compilation. In: proceedings of the 29th ACM international conference on architectural support for programming languages and operating systems, Volume 2. La Jolla CA USA: ACM; 2024s. 929–47. 10.1145/3620665.3640366

[26] Virtanen P, Gommers R, Oliphant TE, Haberland M, Reddy T, Cournapeau D, m.fl. SciPy 1.0: fundamental algorithms for scientific computing in python. Nat Methods. 2020;17(3):261–72. 10.1038/s41592-019-0686-2 PMC7056644 32015543

[27] Pedregosa F, Varoquaux G, Gramfort A, Michel V, Thirion B, Grisel O, m.fl. Scikit-learn: machine learning in python. J Mach Learn Res. 2011;12(85):2825–30.

[28] Jun P, Pagé I, Vette A, Kawchuk G. Potential mechanisms for lumbar spinal stiffness change following spinal manipulative therapy: a scoping review. Chiropractic & Manual Therapies. 2020;28(1). 10.1186/s12998-020-00304-x 10.1186/s12998-020-00304-x PMC7087370 32293493

[29] Kahraman T, Ozcan Kahraman B, Salik Sengul Y, Kalemci O. Assessment of sit-to-stand movement in nonspecific low back pain: a comparison study for psychometric properties of field-based and laboratory-based methods. Int J Rehabil Res. 2016;39(2):165–70. 27031182 10.1097/MRR.0000000000000164

[30] Kulig K, Landel R, Powers CM. Assessment of lumbar spine kinematics using dynamic MRI: a proposed mechanism of sagittal plane motion induced by manual posterior-to-anterior mobilization. J Orthop Sports Phys Ther. 2004;34(2):57–64. 15029938 10.2519/jospt.2004.34.2.57

[31] Nim CG, O’Neill S, Geltoft AG, Jensen LK, Schiøttz-Christensen B, Kawchuk GN. A cross-sectional analysis of persistent low back pain, using correlations between lumbar stiffness, pressure pain threshold, and heat pain threshold. Chiropr Man Therap. 2021;29(1):34. 34479585 10.1186/s12998-021-00391-4 PMC8414715

[32] Breen A, Hemming R, Mellor F, Breen A. Intrasubject repeatability of in vivo intervertebral motion parameters using quantitative fluoroscopy. Eur Spine J. 2019;28(2):450–60. 10.1007/s00586-018-5849-9 30535658

[33] Breen A, Claerbout E, Hemming R, Ayer R, Breen A. Comparison of intra subject repeatability of quantitative fluoroscopy and static radiography in the measurement of lumbar intervertebral flexion translation. Sci Rep. 2019;9(1):19253. 10.1038/s41598-019-55905-1 PMC6917745 31848427

[34] Triano JJ, Budgell B, Bagnulo A, Roffey B, Bergmann T, Cooperstein R, m.fl. Review of methods used by chiropractors to determine the site for applying manipulation. Chiropractic & Manual Therapies. 2013;21(1). 10.1186/2045-709X-21-36 10.1186/2045-709X-21-36 PMC4028787 24499598

[35] Stolz M, von Piekartz H, Hall T, Schindler A, Ballenberger N. Evidence and recommendations for the use of segmental motion testing for patients with LBP—a systematic review. Musculoskelet Sci Pract. 2020;45:102076. 31733430 10.1016/j.msksp.2019.102076

[36] Nolet PS, Yu H, Côté P, Meyer AL, Kristman VL, Sutton D, m.fl. Reliability and validity of manual palpation for the assessment of patients with low back pain: a systematic and critical review. Chiropr Man Therap. 2021;29(1):33. 10.1186/s12998-021-00384-3 PMC8390263 34446040

[37] Nim CG, Downie A, O’Neill S, Kawchuk GN, Perle SM, Leboeuf-Yde C. The importance of selecting the correct site to apply spinal manipulation when treating spinal pain: myth or reality? A systematic review. Sci Rep. 2021;11(1):23415. 34862434 10.1038/s41598-021-02882-z PMC8642385

[38] Hayden JA, Ellis J, Ogilvie R, Stewart SA, Bagg MK, Stanojevic S, m.fl. Some types of exercise are more effective than others in people with chronic low back pain: a network meta-analysis. J Physiother. oktober 2021;67(4):252–62. 10.1016/j.jphys.2021.09.004 34538747

[39] Sørensen PW, Nim CG, Poulsen E, Juhl CB. Spinal manipulative therapy for nonspecific low back pain: does targeting a specific vertebral level make a difference?: A systematic review with meta-analysis. J Orthop Sports Phys Ther. 2023;(9):1–11. 10.2519/jospt.2023.11962 37506306

[40] Nim C, Aspinall SL, Cook CE, Corrêa LA, Donaldson M, Downie AS, m.fl. The effectiveness of spinal manipulative therapy in treating spinal pain does not depend on the application procedures: a systematic review and network meta-analysis. J Orthop Sports Phys Ther [Internet]. 7:2025 10.2519/jospt.2025.12707 39869665

